# 

**Published:** 1818-06

**Authors:** 


### CATALOGUE OF MEDICAL BOOKS.

Sure Methods of attaining a Long and Healthful Life; with the Means of correcting a Bad Constitution. By Lewis Cornaro. Translated from the Italian. Thirty-first edition. 12mo. 2s.

Nosology; or a Methodical Arrangement of Diseases into Classes, Orders, Genera, and Species, accurately defined. From the Latin of William Cullen, M.D. late Professor of Physic in the University of Edinburgh, &c. &c. A new edition. 32mo. 2s.

Practical Illustrations of Typhus Fever and other Febrile and Inflammatory Diseases. By John Armstrong, M.D. Second edition. 8vo. 10s. 6d.

Results of an Investigation respecting Epidemic and Pestilential Diseases, including Researches in the Levant concerning the Plague. By Charles Maclean, M.D. Lecturer on the Diseases of Hot Climates to the Hon. East India Company. 8vo. Vol. 2, 15s.

Surgical Essays. By Astley Cooper, F.R.S. Surgeon to Guy's Hospital; and Benjamin Travers, F.R.S. Surgeon to St. Thomas's Hospital. Part I. Second edition. 8vo. 10s. 6d.

Treatise on the General Principles of Chemical Analysis. Translated from the French of L. J. Thenard, Member of the Institute of France, Professor of Chemistry, &c. &c. By Arnold Merrick. 8vo. 12s.

Letter to the General Court of Contributors of the Royal Infirmary of Edinburgh; containing Observations on the Minutes of Evidence, and on the Report of the Committee appointed to enquire into the State of that House. By a Contributor. Second edition. 1s. 6d.

Second Letter to the General Court of Contributors of the Royal Infirmary of Edinburgh; containing Remarks on the Proceedings at the Meeting held on the 30th March, 1818: to which are annexed, the Report of the Committee, the Reported Speech of the Lord President, and the Resolutions of the General Meeting of the 30th March. 2s.

Report of the Committee of the London Infirmary for curing Diseases of the Eye, occasioned by the False and Calumnious Statements contained in a Letter addressed by Sir William Adams to the Rt. Hon. and Hon. the Directors of Greenwich Hospital. 3s. 6d.

An Attempt to Estimate the Power of Medicine in controling Fever. By W. Brown, M.D. Fellow of the Royal College of Surgeons, and Emeritus Surgeon of the Royal Infirmary. 2s. 6d.

---

### *NOTICES TO CORRESPONDENTS.*

From the Publisher.]—Mr. J. A., *Surgeon, late of Tadcaster, Yorkshire, will oblige the Proprietors of this Journal, by directing some person to settle the account for his Advertisement. The Proprietors are under the necessity of requesting payment through this medium, their letter addressed to Mr. A. on the subject having been returned through the post-office.*

*Several other gentlemen who stand indebted for Advertisements, &c. are requested to settle for the same without delay.*

*The Proprietors take this opportunity to apprize their friends that, in future, all Advertisements must be paid for before insertion.*

From the Editor.]—*Communications are received from* Dr. M'Lean, Dr. Furgeson, Dr. Adams, Mr. Walles, Mr. Brown, B. C. D. *and other anonymous writers, all which shall appear, if possible, in our next. To our eastern correspondent,* Mr. R. *we can only offer the same apology as to our correspondent,* Mr. P. *in our last Number. The work, to which he alludes, is confined to a different set of readers from those of the London Medical and Physical Journal. To notice it would therefore only be a means of complying with the wish of its proprietors, who seem anxious either to procure respectable names in their own work, or to bring themselves into notice without so much expense of advertising; and, above all, to be numbered among the regular established periodical productions.*

### INDEX

### TO THE THIRTY-NINTH VOLUME.

ABDOMEN, Dr. Crampton on the escape of the contents of the stomach into the cavity of the 154
————, Mr. Littleton's cases of pains in the 452
Abdominal viscera, Dr. Perceval's report of certain morbid concretions of the 488
Abercrombie, Dr. on dropsical affections 411
Absorption of bone, remarks on 146
Acid, on the *modus operandi* of nitro muriatic 339
Adams, Dr. his observations on fever 27
————, his letter to the Lord Mayor, on the health of the metropolis 39
————, on muscæ volitantes 103
————, on morbid poisons 209
————, his remarks on Mr. Moore's History of Vaccination 205
————, his answer to Dr. Curry on the claims of John Hunter to the discovery of pus 462
————, Sir Wm. on extraction of the cataract 127
————, his letter to the Directors of Greenwich Hospital on the Egyptian ophthalmia 132
————, extracts from 128
————, Mr. Stevenson's refutation of the charges in his publication 177
————, Dr. Vetch on his treatment of ophthalmia 309
Adder, Dr. Leslie on the bite of an 161
Adhesive powder, Mr. Walker's composition for 162
Air, Dr. Davy on the state of, in the Fever hospitals at Cork 274
Alimentary canal, sometimes mistaken for syphilis 412
Alkalies, Dr. Marcet on the lithontriptic powers of 320
Ammoniaco nitrate of silver, a test for arsenic 28
Amphibia, Dr. John Davy on the urinary organs and secretions of 514
Amputation of the foot, Mr. Hayward's case of 523
Analogous affections, Mr. Lawrence's two cases of 155
ANALYSIS of new publications 45, 114, 223, 304, 395
Anatomy, authors recommended for studying 196
Animal heat, Mr. Hunter on 254
Anti-variolous power of vaccination, Mr. Colville on 160
Aorta, Mr. Samuel Young's remarks on tying the 180
Apoplexy, Dr. Girdlestone's symptoms of, from a large gall-stone 441
Apoplectic diathesis, Dr. Cheyne on the virtues of James's Powder in the 498
Apothecaries, meeting and resolutions of 256
Aretæus, Mr. Wadd on, on elephantiasis 307
Armstrong, Dr. on the scarlet fever, pulmonary consumptions, and chronic diseases 499
Arsenic, Mr. Hume on poisoning with 27
————, preferred in intermittents 58
————, ulcer of the tongue, successfully treated by 76
————, Mr. Kerr's answer to Mr. Hume on tests for 189
————, Mr. Prideaux on the test of silver for 279
————, poisoning by the introduction of, into the vagina 343
Arsenical tests, Mr. Crowfoot's correction of his paper on 376
————, Mr. Hume on the detection of 466
———— applications, cancerous ulceration cured by 518
Arteries, on inflammation of the 7
————, Mr. Lawrence on the ligatures of the 427

INDEX.

Arts, manufactures, and commerce, premiums offered by the Society for the encouragement of 432
Asia, Capt. Burney on the north-eastern part of 253
Atmosphere, Dr. Johnson on the influence of the 247
———, remarks on Sydenham, on the constitution of the 262
Atropy mesenteric, observations on 339
Bailey, Mr. on the use of belledonna in disorders of the head and face 141
Bateman, Dr. on the fever institution 34
Balfour, Dr. on the cure of whitlow by compression 187
Barley, M. Proust's analysis before and after germination 258
Beclard, M. his account of a female dwarf 258
Bedlam, the New, Mr. Frend's remarks on 471
Beer, on the fermentation of 302
Bell, Mr. his remarks on chancre 235
Belladonna, success of, in a case of tic douloureux 176
Bernard, Sir Thomas, extract from his letter to Mr. Vansittart, on the operation of the salt duties 289
———, on the improvement of waste lands 290
———, on salt as manure 291
Biliary ducts, Mr. Todd's case of enlargement of the 499
Blagden, Mr. his case of fatal hæmorrhage from the extraction of a tooth 76
Blaine, Mr. his treatise on the diseases of dogs commended 5
Blood, Mr. Hunter on the vitality of the 254
Blow-pipe, Mr. Watt's improved method of using the 520
Bones, Mr. Howship on the morbid structure of 145
———, ossific matter upon the surface of 147
———, observations on the diseases of 148
———, on the swelling of 149
Books, retrospective view of new medical 525
———, catalogue of medical 88, 176, 264, 349, 440, 528
Bots, in horses, certain remedies for 370
Bourdillon, the Rev. T. his letter on inoculation 337
Boy, report of a, born deaf and dumb, recovering 179
———, Mr. Curtis on his case 256
Brain, on its sympathetical affection for the diseased part 4
———, observations on the 53, 373
Brain, Dr. Kinglake on concussions of the 457
Brandish, Mr. his case of urinary calculus 449
Breen, Dr. on the practice of midwifery 159
Brine, Dr. on the health of the metropolis 168
Bristol, extract of a letter from, on mesenteric atropy 339
Brown, Dr. on blood-letting from the hæmorrhoidal veins 406
Burrows, Dr. on the medical care of the poor 332
Butter, Mr. on the effects of nitre 160
Cæsarian operation, observations on 13
Calculous disorders, Dr. Marcet on 313
Calculus, urinary, Mr. Brandish's case of 448
Calabrian ash tree, Dr. Prout on the liquor oozing from the leaves of 326
Canada, Dr. Swediaur's account of cutaneous disease in 209
———, account of an extensive infection in 209
Cancerous ulceration, cured by arsenical applications 518
Carpue, Mr. his success in the Taliacotian operation for restoring the nose 16
Cases—extraordinary instance of the petrifaction of the heart, 7; palpitation of the heart, 9; aneurism of the aorta, 10; on the urethra, 12; contraction of the uterus, 13; perforation of the diaphragm, 15; obstinate costiveness, 17; the extinction of fever in Baron Trenck, from swallowing cold water, 56; examination of a dead body after gastric fever, 61; dissection after choleric, ib.; after dysenteric, ib.; of lusus naturæ, 67; of hernia cerebri, 71; motions of the brain, 74; ulcer upon the tongue, 76; lithotomy, ib.; fatal hæmorrhage from the extraction of a tooth, 76; remarkable symptoms connected with a painful affection of the extremity of the thumb, 77; Miss M'Avoy, 84; tabes mesenterica, 92; a damson in the trachea, 100; an ear of bearded wheat in the trachea, 102; wound in the head, ib.; muscæ volitantes, 103, 107; ophthalmic form of fever, 111; Miss M'Avoy, 114; the keratonyxis, 134; cataractæ adhærentes capsulares, 139; disorders of the head and face, 142; hemicrania, 144; tic douloureux, success of Belladonna in it, 176; spina bifida, 183; of whitlow, 186; fœtus with external hydrocephalus, 197; lues venerea netha seu spuria, 206; eruptions from an unknown venereal affection, 220; sulphurous fumigations against inveterate venereal eruptions, 221; periodical hypochondriasis, 249; of fractured humerus, 256; application of fire in the cure of sciatica, 257; an American girl, deaf, dumb, and blind, 265; neuralgia of the facial nerve, 282; ophthalmia, 309; poisoning by the introducing arsenic into the vagina, 343; on a wound on the testicle, followed by the formation of a bony concretion, 344; phenomena of vision, 359; muscæ volitantes, 361; continued fever, 362; diabetes mellitus, 366; of worms from want of salt, 369; of stuttering, 377; hooping-cough, 383; of gun-shot wounds of the heart, 404; of a disease producing hydrothorax and anasarcous dropsy, with constant expectoration, 405; calculi removed from the urethra, 417; extra-uterine fœtus contained in the fallopian tube, 429; of long continued serous discharge per vaginam, 438; symptoms of apoplexy from a large gall-stone, 441; placental presentation, with excessive hæmorrhage, in the seventh month, 443; urinary calculus, 449; spasms of the stomach, ib.; convulsion, 451; pains in the abdomen, ib.; ischuria, 454; that of Miss M'Avoy, 474; symphilitic inflammation, 477; poisoning by a decoction of black hellebore, 480; autumnal fever, 487; contagion, 491; petechial febricula, 493; from taking two ounces of the camphorated tincture of opium, in mistake for a purgative draught, 495; melæna, ib.; of jaundice, 496; dropsy—enlargement of the biliary duct, 499; epidemic small-pox, 509; pulmonary consumption, 511; peronitis, 513; mal-conformation of the uterine system in women, 514; cancerous ulceration, 518; iritis, ib.; foreign substance lodged in the lungs, 522; amputation of the foot, 523

Cataract, Sir William Adams on the extraction of the 127
———, M. Roux on the operation for the 515
Cattle, advantages of salt to 292
Caustics and mercurials, Dr. Janin on the use of, in syphilis 477
Chancre, Mr. Bell's remarks on 235
Chemistry, animal and vegetable, observations on 16
———, authors recommended for studying 194
Cheyne, Dr. his report of the Hardwicke fever hospital 483
———, his case of melæna 495
——— ——— jaundice 496
———, on the virtues of James's powder in the apoplectic diathesis 498
Children, review of Dr. Davis's enquiry into the causes of mortality among 70
Chrestien, Dr. on the medical properties of the preparations of gold 515
Chronic diseases, Dr. Francis Moore on 169
———, Dr. Armstrong on 499
Climate of England, on the deterioration of the 522
Cloquet, M. his case of fœtus, with external hydrocephalus 197
Club-feet, Dr. Colles on the cure of 489
Coal-owners of the Tyne, service of plate presented by, to Sir Humphry Davy 84
——— mine, explosion of a 340
Cogan, Dr. Thomas, memoir of 346
Colchicum, dispute between Messrs. Want and Wilson concerning the preparation of 81

Collectanea Medica, 34, 107, 201, 288, 375, 471
Colles, Dr. on the cause of trismus nascentium 497
Colville, Mr. on the anti-variolous power of vaccination 160
Contagion, Dr. Goodison's case of 491
Contagions, observations on 44
Convalescence, Dr. Jackson on the danger of returning inflammation in a state of 59
Convulsion, Mr. Littleton's cases of 451
Cooper, Mr. Samuel, review of his History of a Case of Lithotomy 76
——, Mr. Astley, Mr. Young's remarks on the case of aorta in his Essays 182
————, his three cases of calculi removed from the urethra 417
Corfu, Dr. Goodison on the plague of 491
Cork, state of the health of 83
——, Dr. Davy on the state of air in the fever hospitals at 274
Cornea, opaque, Dr. Vetch on 310
Correspondents, notices to 88, 176, 264, 352, 440
Cornwall, distress of the poor in, from want of salt 368
Costiveness, obstinate, a case by Mr. Reeve 17
Cotton and wool manufactures, queries concerning the health of children engaged in the 340
Cotton manufactories, Dr. Jackson on, affecting the health 464
Crichton, Dr. his experiments with the vapour of boiling tar, in the cure of pulmonary consumption 511
Crowfoot, Mr. on arsenical tests 336
Crawfurd, Mr. on the effects of piper-cubeba in gonorrhœa 159
Crampton, Dr. on the rupture of the stomach, and the escape of its contents into the cavity of the abdomen 154
Croft, Sir Richard, memoir of 259
Curry, Dr. James, on the health of the metropolis 37
————, letter from, with remarks of the editor 79
————, his letter on pus 353
————, Dr. Adams' answer to, on the discovery of pus 462
Curtis, Mr. on the case of a boy born deaf and dumb recovering 256
Cutaneous disease in Canada, Dr. Swediaur's account of 209
Cuvier, extract from the annual report of 516
Davis, Dr. review of his Enquiry into some of the principal causes of the Mortality among Children 70
Davy, Sir Humphry, service of plate presented to, by the coal-owners of the Tyne 84
——, Dr. John, on the state of air in the fever hospitals at Cork 274
————, on the urinary organs and secretions of amphibia 514
————, on the remedies used at Ceylon against the bites of venomous serpents 284
Denman, Dr. some account of 259
————, anecdote of 261
Dewar, Mr. his account of an epidemic small-pox in Cupar in Fife 409
Diabetes, explanation of phenomena of 322
————, Dr. Trotter on 366
Diamond, Professor Jameson on the natural history of the 432
Digestion, M. Montegre on 2
Digitalis, Dr. Uwins on the medical effects of 91
————, uncertainty in the action of 94
Disease, Dr. Prout on urine, and its changes under 322
————, enquiry respecting a doubtful 337
————, Dr. Pearson's case of a, producing hydrothorax and anasarcous dropsy, with constant expectoration 405
Diseases, report of, 86, 176, 262, 478, 526
Dissection, observations on 62
Dog and a man, Mr. Young on the analogy betwixt, in tying up the aorta of a 183
Dovar, Dr. his remarks on opium 448
Dropsy and tabes mesenterica, analogy of 93
————, Dr. Perceval on 498
Dropsical affections, Dr. Abercrombie on 411
Dry rot in ships, remarks on the 433
Dublin hospitals, reports of 482
Duchateau, his remedy for palpitation of the heart 9
Dundas, Sir David, his Hunterian oration 253
Durham, explosion of a coal mine in the county of 340
Dysentery, remarks on 154

Ear, Mr. W. Wright's essay on the human 139
———— extract from ib.
Edinburgh, extracts from the Medical and Surgical Journal 155, 240, 404
———— university, course of medical education at 192
Editor, his remarks on the bills of mortality 172
Education, of a physician, enquiry respecting 80
————, medical, on conducting a 192
Edwards, Mr. G. F. his case of placenta presentation 443
Elephantiasis Italica, Mr. Good's observations on 216
———— Mr. Wadd on 307
———— Mr. Wadd on Aretæus on 26
Endemic diseases, observations on 43
English, Abbé Raynal's remarks on the 340
Epidemic diseases, observations on 44
Epidemic petechial febricula, Dr. Percival on an 493
Experiments, Dr. Davy on air 274
———— on refuse salt 289
————, in dissection of dead bodies 330
————, Dr. Porterfield on spectra 357
————, Sir E. Home on the passage of the ovum 395
———— on snails' mode of breeding 400
———— on the glands of the human stomach 402
———— Dr. Wood's, on urine 455
———— in a case of poisoning by black hellebore 480
———— dissection after autumnal fever 487
————, Dr. Crichton's, made with the vapour of boiling tar, in the cure of pulmonary consumption 511
———— on a living guinea-pig 518
Face, Mr. Bailey on the use of belladonna in disorders of the 141
Febrile diseases, review of Mr. Jackson's history and cure of 45
———— extracts from 46
———— origin, Dr. Kinglake on 37
Female dwarf, M. Beclard's account of 258
Ferguson, Dr. his enquiry into the origin and nature of the yellow fever 149
Fever Institution, Dr. Bateman on the 34
————, Dr. Brine on the 168
————, on the, among the poor ib.
————, the suppurative process 49
————, on the, in the West Indies 51
————, the cause of 54
————, on the mercurial plan of treating the 57
————, examination of a dead body after gastric 60
————, dissection after choleric 16
————, after dysenteric ib.
————, ophthalmic form of, Dr. Jackson on 111
————, yellow, Dr. Ferguson's enquiry into the origin and nature of the 149
———— hospitals at Cork, Dr. Davy on the state of the air at the 274
————, Mr. Syer on, continued 362
————, a case of ib.
————, on the true nature of 372
————, typhus, Mr. Kidd's account of, in Ireland 408
————, remittent, Dr. Goodison's observations on 491
Fevers, extract from Sutton on 485
Fire, Mr. Potet on the application of, in the cure of sciatica 257
———— balls, meteors, Dr. Maskelyne's plan for observing 341
Fœtus, with external hydrocephalus, M. Cloquet's case of 197
————, Mr. Langstaff's case of extra uterine, contained in the fallopian tube 429
Fordyce, Sir William, observations on his cases of venereal 241
Fowler, Dr. Mr. Home's observations on his *solutio arsenicalis* 31
Frend, Mr. on the smoke of London 471
Frog, Mr. J. Wilson on the eggs of the common 432
Fuge, Mr. his case of gun-shot wounds in the heart 404
Fungus hæmatodes, Mr. Langstaff's cases of 155
Gamage, Dr. on the hooping-cough 383
Gangrene, the hospital, observations on 14
————, observations on 203
Gastric glands of the human stomach, Sir E. Home on 402

Girdlestone, Dr. his account of symptoms of apoplexy from a large gall-stone 441
Girl, deaf, dumb, and blind, account of a 279
Gold, Dr. Chrestien on the medical properties of the preparations of 515
Gonorrhœa, Mr. Crawfurd on the effects of piper cubeba, in 159
———— benigna, new injective for 176
————, extract from Mr. Hunter's treatise on, on matter formed without ulceration 286
Good, Mr. and Dr. Parr, their account of the pellagra 216
————, on elephantiasis italica ib.
Goodison, Dr. his observations on remittent fever, and on the plague of Corfu 491
Gout, Dr. James Johnson on 250
——, Sir E. Home on the use of colchicum autumnale in 400
Grant, Dr. his remarks on synochus putris 483
Granville, Dr. on the mal-conformation of the uterine system 514
Greenwich-hospital, Sir William Adams' letter to the directors of, on the Egyptian ophthalmia 126
Gum, M. Proust's remarks on 298
Guthrie, Mr. on syphilis 237
Hæmorrhage, fatal, Mr. Blagden's case of, from the extraction of a tooth 76
Hæmorrhoidal veins, Dr. Brown on blood-letting from 406
Hardwicke fever-hospital, Dr. Cheyne's medical report of 483
Hayward, Mr. his case of amputating the foot 523
Health, Dr. Jackson on cotton manufactories affecting the 465
Heart, Mr. Fuge's case of gun-shot wounds of the 404
————, Mr. James on the nature and causes of diseases of the 418
Hartley, Dr. on sensation and motion 98
Hayes, Dr. Pliny, his case of spina bifida 183
Head, Baron Larrey's case of a wound in the 102
——, Mr. Bailey on the use of belladonna in disorders of the 141
Heart, on the diseases of the 7
Heberden, Dr. on affections of the prostate 314
————, on stone ib.
Hellebore, black, on poisoning produced by 481
Hermaphrodite, account of a 519
Herni crania, case of 144
Hernia cerebri, Mr. Stanley's cases of 71
Hennen, Mr. on the cure of syphilis, and on an affection of the alimentary canal, mistaken for syphilis 412
————, extracts from 72
Hints to credulity, review of Mr. J. Sanders' tract, entitled, "on the Case of Miss M'Avoy" 114
Holland, Dr. his account of the pellagra 211
Home, Sir Everard on the passage of the ovum from the ovarium, to the uterus in women 395
————, on the use of colchicum autumnale in gout 400
—— on the ova of the sepia, and the vermes testacea ib.
—— some account of the nests of the Java swallow, and of the glands that secrete the mucus 401
—— on the glands of the human stomach 402
Hooping-cough, Dr. Gamage on the 383
————, Dr. Watt on ib.
————, cases of 385
————, remarks on 438
Howship, Mr. on the morbid structure of bones 145
Human mind, Dr. Parkinson's analysis of the 96
————, the elements of the 97
Hume, Mr. on poisoning with arsenic 27
————, Mr. Kerr's answer to 189
————, on the detection of arsenic 466
Humerus, M. Odienne, his case of fractured 256
Hunter, John, on his system of physiology 2
————, eulogium on, at the French Institute 85
————, on the swelling of bone 149
————, on his reading 223
————, on the venereal chancre 228
————, on the introduction of the venereal disease at Otaheite 229
————, his doctrine of morbid poisons confirmed 231
————, observations on 243
————, his theory of the vitality of the blood 254

Hunter, John, on animal heat ib.
——————, on microscopical evidence 397
Hunter, Dr. William, on his claims, and Dr. Morgan's, to pus as a secretion 285
Hydrocephalus and tapes mesenterica, connection of 95
Hydrocephalus internus, hints on the increase of 470
Hypochondriasis, periodical, Dr. Johnson's case of 249
Hygëia arthritica, Dr. Johnson on 251
Infection, observations on 44
——————, remarkable instance of 86
Infirmaries, on the propriety of building 334
Inflammation, Dr. Jackson on the danger of its returning, during convalescence 59
——————, Dr. Hunter on matter formed by, without ulceration 286
Inflammatory diseases, remarks on 373
Inoculation, the Rev. Mr. Bourdillon's letter on 337
Insanity, observations on 4
——————, treatment of 483
Intelligence, medical and philosophical, 79, 161, 252, 335, 427, 513
Ireland, Mr. Kidd on the typhus fever prevalent in 408
Ischuria, Dr. Wood's case of 454
Isiris, case of 518
Itard's, M. his memoir on stuttering 375
Jackson, Dr. review of his history and cure of febrile diseases 45
——————, on ophthalmic form of fever 111
——————, on cotton manufactories affecting the health 464
James, Mr. his inquiry into the nature and causes of diseases of the heart 418
James's Powder, Dr. Cheyne on the virtues of, in the apoplectic diathesis 498
Jameson, Dr. on the natural history of the diamond 432
Janin, Dr. on the use of caustics and mercurials in syphilis 477
Jaundice, Dr. Cheyne's case of 496
Java swallow, Sir E. Home's account of the nests of the 401
Jenner, Dr. extract from Mr. Moore on his vaccine discovery 266
Johnson, Dr. James, on the influence of the atmosphere 247
——————, his case of periodical hypochondriasis 249
Johnson, Dr. James, on gout 250
——————, on the hygëia arthritica 251
——————, on rheumatism ib.
Keratonyxis, M. Mensert on the operation of 135
——————, observations respecting the 137
Kerr, Mr. his answer to Mr. Hume on tests for arsenic 189
Kidd, Mr. his account of the typhus fever prevalent in Ireland 408
Kinglake, Dr. on febrile origin 371
——————, on depressed vital action 457
Landaff, the Bishop of, his method of cure for the stone 317
Lane, Mr. Charles, review of his history of a case of ulcer of the tongue 76
Langstaff, Mr. his cases of fungus hæmatodes considered 155
——————, his case of extra-uterine fœtus contained in the fallopian tube 429
Larrey, Baron, his case of a wound in the head 102
Laryngotomy, Mr. Whitley on the operation of 101
Lawrence, Mr. his cases of analogous affections considered 155
Lectures announced 85, 261, 350, 437, 525
Lemercier, Dr. on a neuralgia of the facial nerve 283
Leprosy, method of cure of 207
Leslie, Dr. on the bite of an adder 161
Lightning on a tree, effect of 81
Linseed cataplasms, Mr. Walker on 162
Liverpool, blind asylum at 115
Lithotomy, review of Mr. Samuel Cooper's History of the case of 76
——————, Mr. Smith's case of 408
Littleton, Mr. on some phenomena of vision 354
—————— on the utility of large doses of opium 447
——————, his cases of spasms of the stomach 450
Locke, Mr. Abbé Raynal's remarks on 341
London, Transactions of the Medical Society of 171, 335
Lues venerea notha seu spuriá, remarks on the 206
Lungs, a case of a foreign substance lodged in the 522
Lusus Naturæ, review of Dr. Sanctis' tract entitled 67

M'Avoy, Miss, remarks on the case of 84
————, Dr. Renwick's Narrative of 115
————, Mr. J. Sandars's Hints to Credulity, occasioned by ib.
————, further remarks on 163
————, Dr. Renwick on her case 474
Machell, Mr. T. his portable inhaler and topical vapour-bath 89
Magendie, M. observations on his system of physiology 2
————, his report to the Royal Academy of Sciences, on the excellency of prussic acid in cases of phthisis 81
Magnesia, Dr. Trotter's successful treatment of diabetes mellitus by 266
Man and a dog, Mr. Young on the analogy betwixt the tying up the aorta of a 183
Manna, from the Calabrian ash-tree, Dr. Prout on 326
Manoury, Dr. on a wound of the testicles 344
Marcet, Dr. on calculous disorders 313
Marshall, Mr. on the medical virtues of salt 368
Maskelyne, Dr. his plan for observing the meteor called fire-balls 341
Materia Medica, studies recommended for the 194
Matters, Mr. Smithson, on the colouring properties of certain 253
Mead, Dr. extract of a letter to, on diseases of the skin and genitals 205
Medical and Philosophical Intelligence, 79, 161, 252, 335, 427, 513
Medicine, historical retrospect of recent improvements in 1
————, zootic, observations on 5
————, authors recommended for the practice of 194
————, Dr. Uwins' oration on the theory and practice of 335
Medico-Chirurgical Transactions, account of 71, 145, 223, 322, 417
Melæna, Dr. Cheyne's case of 495
Mensert, M. on the operation of keratonyxis 134
Meteorological Journal kept in London 87, 175, 263, 351, 439, 527
Meteors, called fire-balls, Dr. Maskelyne's plan for observing 341
Metropolis, letters on the health of 34, 167
Microscopical evidence, John Hunter on 397
Midwifery, Dr. Breen on the practice of 159
Milk, Mr. Sutcliffe's case of peritonitis relieved by injections of warm 513
Montegre, M. on digestion 2
Moore, Mr. letter to, on vaccination 20
————, Dr. Adams's remarks on his history of vaccination 265
————, Dr. Francis, on ophthalmia 169
Morbid poisons, observations on 13
————, extracts from Dr. Adams on 203, 271
Morgan, Mr. on the health of the metropolis 169
————, Dr. on the claims of, and Dr. Wm. Hunter, to the theory of pus as a secretion 285
Mortality, bills of, for 1817 172
Murray, Dr. on definite proportions in chemical combinations to the constitution of acids, alkalies, and earths, and their compounds 431
Muscæ volitantes, Dr. Adams on 103
————, Mr. Ware on 105, 106
Nerve, facial, Dr. Lemercier, on a neuralgia 282
Nervous persons, Mr. Ware on the muscæ volitantes of 106
Nitre, Mr. Butter on the effects of 160
Nosology, observations on 3
Nosological observations, Dr. Parkinson's 461
Olive, cultivation of the 519
Opium, a specific in the venereal disease 207
————, Mr. Littleton on the utility of large doses of 447
————, Sydenham on 448
————, Dr. Dovar on ib.
Ophthalmia, Egyptian, observations on 133
————, Dr. Vetch on Sir Wm. Adams's treatment of 308
Ophthalmic form of fever, Dr. Jackson on 111
Orang outang, Dr. Trail's account of an African 431
Otaheite, Mr. Hunter on the introduction of the venereal disease into 229
Ovum, Sir E. Home on the passage of the, from the ovarium to the uterus in women 395
Paraphymosis, Mr. Wadd on 305
Paris, report of the royal hospitals of 436

Parkinson, James, his treatise on the shaking palsy commended 5
———, Dr. his analysis of the human mind 97
————, his nosological observations 460
Parturition, efficacy of sweet oil previous to 80
Pathology, English, observations on 3
———, zootic 6
———, inferiority of the French in 10
———, premiums offered by the Society of Arts, Manufactures, and Commerce, for subjects connected with 432
Paul, St. a regulation of the school of, in the sixteenth century 343
Pearson, Mr. his account of remarkable symptoms connected with a painful affection of the extremity of the thumb 77
———'s, Dr. instance of a disease producing hydrothorax and anasarcous dropsy, with constant expectoration 405
Pellagra, Dr. Holland's account of the 211
———, Dr. Good's and Dr. Parr's account of 217
Percival, Dr. on an epidemic petechial febricula 493
————, on the medicinal effects of green tea 495
————, on dropsy 499
————, his report of certain morbid conditions of the abdominal viscera 488
Periosteum, M. Cloquet's case of dropsy of the 197
Peritonitis, Mr. Sutcliffe's case of 513
Pharmacy, authors recommended for studying 194
Phenomena, singular, in Baron Larrey's case of a wound in the head 12
Phlegmasia dolens, observations on 12
Phthisis, Dr. Magendie on the excellency of Prussic acid as a medicine in cases of 81
Phymosis, Mr. Wadd on 305
Physic, on the fundamental principles for regulating the profession of 155
Physician, enquiry respecting the academic education of a 80
Physicians, lectures at the College of 518
Physiology, observations on 2
———, authors recommended for studying 195
Physiologist, advice to the rural 297
Piper cubeba, Mr. Crawfurd on the effects of, in gonorrhœa 159
Pitcairn and Erasmus on the universality of syphilis 219
Placenta presentation with excessive flooding, Mr. Edwards's case of 443
Poison, Mr. Hume on, with arsenic 27
Poor, on the fever among the 34
——, instances of the mal operation of the salt-duties, on the 293, 297
——, parochial, Mr. Yeatman's remarks on the medical care of the 331
————, Dr. Burrows on 332
Portable inhaler and topical vapour-bath, Mr. T. Machell's 89
Portal, M. and M. Girard's remarks on vomiting 517
Portugal, treatment of the venereal disease in 228
Potet, M. on the application of fire in the cure of sciatica 257
Prepuce, cancerous, Mr. Wadd on 306
Prideaux, Mr. on the test of silver for arsenic 297
Private parts, on diseases of the 201
Prostate, Dr. Heberden on the affections of the 314
Proust, M. his analysis of barley before and after its germination 297
Prout, Dr. on urine and its changes under disease 322
————, on the Calabrian ash-tree 326
Prussic acid, Dr. Magendie on the excellency of, in cases of phthisis 81
Pulmonary consumption, Dr. Armstrong on 499
————, Dr. Crichton's experiments made with the vapours of boiling tar in the cure of 511
Pus, as a secretion, on the claims of Dr. Morgan and Dr. William Hunter to the theory of 285
——, Mr. Wadd on 305
——, Dr. Curry on 353
——, Dr. Adams's answer to Dr. Curry on the discovery of 462
Raynal, Abbé, his remarks on the English 340
Remittent fever, Dr. Goodison's observations on 491
Renwick, Dr. review of his narrative of the case of Miss M'Avoy 114
————, his further account of her 164
————, on Miss M'Avoy's case 474

Resin, M. Proust's remarks on 298
Rheumatism, Dr. James Johnson on 251
Rose, Mr. review of his observations on the treatment of syphilis without mercury 224
Rot among sheep, remarks on 433
Roux, M. on the operation for the cataract 515
Rowe, Mr. a case by, of obstinate costiveness 17
———, his observations on fever 39
Royal Society of London, annual meeting of the 252
———, philosophical transactions of 395
———, proceedings of the 430, 514
——— of Edinburgh 430
——— of Paris 514
——— hospitals, report of 434
Salt-duties, on the operation of the 289
———, on the advantages of their removal 294
—— as manure, Sir Thomas Bernard on 291
——, advantages of, to cattle 292
——, Mr. Marshall on the medical virtues of 368
——, preventive for the rot among sheep 434
Sanctis, Dr. review of his tract Lusus Naturæ 67
———, extract from 68
Sandars, Mr. J. review of his Hints to Credulity, in the case of Miss M'Avoy 114
Scarlet-fever, Dr. Armstrong on 499
———, Dr. Willan on ib.
Sciatica, M. Potet on the application of fire in the cure of 257
Schmidt, Dr. on the pathology of the spleen 239
Sensation and motion, Dr. Hartley on 98
Serpents, Dr. Davy's chemical examination of remedies at Ceylon against bites of venomous 284
Shaking palsies, observations on 5
Sheep, the rot among 433
Ships, on the dry rot in ib.
Skin and genitals, on diseases of the 201
Small-pox Hospital, report of the committee of the 81
———, observations on the 271
———, Mr. Dewar's account of an epidemic small pox in Cupar, in Fife 509
Smith, Dr. his case of lithotomy 408
Smithson, Mr. on the colouring properties of certain matters 253
Smoke of London, Mr. Frend's remarks on the 471
Snails, their mode of breeding 400
Solar spectrum, on the magnetizing power of the violet rays of the 524
Spectra, Dr. Porterfield's experiment from 357
Spider, M. Vicat's account of a worm found in the belly of a 478
Spina bifida, Dr. Pliny Hayes's case of 183
Spleen, Dr. C. L. Schmidt on the pathology of the 239
Spurzheim, Dr. his definition of insanity 4
Stanhope, Earl, efficacy of the portable inhaler upon 90
Stanley, Mr. review of his cases of hernia cerebri 71
Starch, M. Prout's remarks on 301
Stevenson, Mr. his refutation of Sir William Adams's charges 177
Stomach, Dr. Crampton on the nature of 154
Stomach, Mr. Littleton's cases of spasms of the 450
Stone, Dr. Heberden on the 314
——, Bishop of Landaff's method of cure for the 319
Stuttering, M. Itard's memoir on 375
———, cure for 379
Sugar, M. Proust's remarks on 298
Surgeons, meeting of the College of 253
———, lectures at the College of 518
———, bill in parliament on 254
Surgery, authors recommended to students in 195
———, Mr. Wadd's cases of 304
Sutcliffe, Mr. his case of peritonitis 513
Sutton, Dr. extract from, on fevers 485
Swediaur, Dr. his account of a cutaneous disease 209
Sweet oil, efficacy of, previous to 80
Sydenham, remarks on the constitution of the atmosphere 262
———, Dr. his remarks on opium 448
Syer, Mr. on continued fever 362
Synochus putris, Dr. Grant on 483
Syphilitic inflammation, Dr. Janin on the use of caustics and mercurials in 477
Syphilis, Pitcairn and Erasmus on the universality of 219
———, French mode of curing 221

Syphilis, Mr. Rose on the cure of without mercury 224
———, Mr. Guthrie on 236
———, Dr. Thompson on 290
Tabes mesenterica and dropsy, analogy of 93
——— ——— and hydrocephalus, connexion of 95
Tar, Dr. Crichton's experiments made with the vapour of boiling, in the cure of pulmonary consumption 511
Taunton, Mr. his report of the London Truss Society 170
Tea, Dr. Percival on the medicinal effects of green 495
Teissier, M. experimental results by, on the gestation of female domestic animals 6
Testicle, Dr. Manoury on a wound of the, followed by the formation of a bony concretion 344
Thompson, Dr. on the cure of syphilis 240
Thumb, Mr. Pearson's account of remarkable symptoms connected with a painful affection of the extremity of the 77
Thunder, on the effects of 174
Tic douloureux, success of belladonna, in a case of 176
———, observations on 283
———, cured by tar 519
Todd, Mr. his case of enlargement of the biliary ducts 499
Tooth, case of fatal hæmorrhage, by Mr. Blagden, by the extraction of a 76
Trail's, Dr. account of an orang-outang 431
Travers, Mr. Young's remarks on the case of aorta in his and Mr. Astley Cooper's essays 186
Trachea, Mr. Whitley's case of a damson-stone in the 100
———, case of an ear of bearded wheat in the 102
Tree, effect of lightning on a 81
Trenck, Baron, extinction of fever in, by cold water 56
Trismus nascentium, Dr. Colles on the cause of 497
Trotter, Dr. on diabetes 366
Truss Society, Mr. Taunton's report of the 170
Tympanum, on the ill effects of perforating the 16
Ulcer of the tongue, review of Mr. Charles Lane's history of a case of 76
University lectures, disadvantages of attending 193
Urethra, Mr. Astley Cooper's cases of calculi, removed from the 417
Urine, Dr. Prout on 322
———, Dr. Wood's experiments and remarks on 455
Uterine system, Dr. Granville on the mal-conformation of the, in women 514
Uterus, Sir E. Home on the passage of the ovum from the ovarium to the, in women 395
Uwins, Dr. on the medical effects of digitalis 91
———, his oration before the Medical Society on the theory and practice of medicine 335
Vaccination, letter to Jas. Moore, esq. on 20
———, the impropriety of unprofessional persons practising 21
———, Mr. Colville on the anti-variolous power of 160
———, Dr. Adams's remarks on Mr. Moore's History of 265
Vagina, case of long-continued serous discharge by the 438
Vansittart, Mr. extract from Sir Thomas Bernard's letter to, on the operation of the salt-duties 239
Vena saphena, on the division of 516
Venereal disease, opium cure for the 207
———, treatment of, in Portugal 228
———, Mr. Hunter on 247
Venereal eruptions, on sulphureous fumigations of 221
——— cases, observations on Sir Wm. Fordyce's 241
Vetch, Dr. on Sir Wm. Adams's treatment of ophthalmia 209
Vicat, M. his account of a worm found in the belly of a spider 479
Vision, Mr. Littleton on the phenomena of 354
Vital action, Dr. Kinglake on depressed 457
Vomiting, M. Portal and M. Girard's remarks on 517
Wadd, Mr. review of his cases of surgery 304
Walker, Mr. on linseed cataplasms 162
———, his composition for adhesive powder ib.
Want and Wilson, Messrs. dispute between, on the preparation of colchicum 81

Ward, Mr. on the *modus operandi* of nitro-muriatic acid 339
Ware, Mr. on muscæ volitantes 103
Waste lands, Sir Thomas Bernard on the improvement of 290
Water, cold, on the affusion of, in fever 56
——— spout in the Isle of Wight, account of 434
Watt, Dr. on the hooping-cough 383
Wernerian Society, proceedings of 481
White's Natural History of Selborne, extract from, on leprosy 208
Whitley, Mr. his case of a damson-stone in the trachea 100
————, on the operation of laryngotomy 101
Whitlow, Dr. Balfour on the cure of, by compression 185
————, remarks on the treatment of 273
Willan, Dr. on scarlet fever 499
Wilson, Mr. on the eggs of the common frog 432
Wine, injection of red port and water in the case of gonorrhœa benigna 176
Women, Sir E. Home on the passage of the ovum from the ovarium to the uterus in 395
———, Dr. Granville on the mal-conformation of the uterine system in 514
Wood, Dr. on the treatment of whitlow 273
————, his experiments and remarks on urine 455
Worm, M. Vicat's account of a, found in the belly of a spider 478
Wound in the head, Baron Larrey's case of a 102
Wright, Mr. his essay on the human ear 139
Yeast, M. Proust's remarks on 303
Yeatman, Mr. on the medical care of the poor 331
Yellow-fever, Dr. Ferguson's enquiry into the origin and nature of the 149
Young, Mr. Samuel, his remarks on tying the aorta 180

Jas. Adlard and Sons, Printers,

23, Bartholomew-Close.

